# Summarising 40 years of gastric lavage studies to evaluate efficiency and survival in sharks and rays

**DOI:** 10.1111/jfb.70006

**Published:** 2025-03-04

**Authors:** Jaelen Myers, Marcus Sheaves, Adam Barnett

**Affiliations:** ^1^ James Cook University Townsville Douglas Queensland Australia; ^2^ Biopixel Oceans Foundation Smithfield Australia

**Keywords:** diet, elasmobranch, gut flush, non‐lethal, recapture, stomach content

## Abstract

Traditionally, lethal stomach dissection has been used to study the diets of sharks and rays, but conservation and animal welfare concerns necessitate non‐lethal alternatives, such as gastric lavage (stomach or gut flushing). In this study, we summarised gastric lavage studies on elasmobranchs to identify which species/groups it has been effective for, the difficulties encountered and if post‐release survival has been evaluated. Secondly, we used a field study to (1) demonstrate how to perform gastric lavage on juvenile rays, (2) assess its effectiveness and (3) verify post‐release survival using mark‐recapture techniques. Only 23 published studies have used gastric lavage on either sharks or rays, indicating that this technique is still highly underutilised in ecological research. Effectiveness at obtaining stomach contents varied but often exceeded 50%, particularly for rays. Captivity studies provided greater evidence of survival than field studies, and only one field study assessed long‐term survival using tag‐recapture methods. In this field study, gastric lavage was highly effective for young juvenile rays, and recaptures verified survival for various periods after release. More research is needed to adapt gastric lavage across a wider range of species and sizes, especially larger sharks. Furthermore, incorporating approaches to validate survival following non‐lethal handling procedures will be essential to ensure ethical compliance and optimal outcomes for research and conservation.

## 1 INTRODUCTION

Stomach content analysis (SCA) is a fundamental technique for studying the feeding ecology of fish (Amundsen & Sánchez‐Hernández, 2019; Hyslop, 1980). It is traditionally performed by euthanizing an animal to dissect its stomach and represents a snapshot of the overall diet (Baker et al., 2024). Historically, lethal SCA has been the primary method for describing the diets of sharks and rays, with sample sizes ranging from hundreds (Dale et al., 2011; Lowe et al., 1996; O'Shea, Thums, et al., 2013; Wetherbee et al., 1997) to over 1000 individuals (Barnett et al., 2013) for a single study. To maximise information collected from euthanised animals, biological and life‐history parameters have also been obtained, such as reproduction or age–growth relationships (Awruch et al., 2009; Lucifora et al., 2009).

Nowadays, researchers are less inclined to kill large numbers of sharks and rays to collect biological or dietary data, despite the useful knowledge that may be gained from this (Heupel & Simpfendorfer, 2010). In addition to the welfare of individual animals, conservation concerns have also put lethal sampling into question, particularly where populations might be negatively impacted if individuals are removed. With many elasmobranchs now vulnerable to extinction, it is harder to justify “killing for conservation” (Hammerschlag & Sulikowski, 2011). Nonetheless, studying the diet has several implications for conservation and fisheries management, such as providing knowledge of predation pressure on fisheries species or identifying essential feeding habitats that support survival and recruitment (Barnett et al., 2017; Barnett & Semmens, 2012; Galván‐Magaña et al., 2019).

Where these studies are necessary, we cannot simply reduce the numbers of animals used without compromising data quality, since large sample sizes are often required to accurately describe diets over time (Kamler & Pope, 2001), particularly for species with broad dietary niches, such as the broadnose sevengill shark, *Notorynchus cepedianus* (Barnett, Abrantes, et al., 2010; Ebert, 2002), or tiger shark, *Galeocerdo cuvier* (Dicken et al., 2017; Lowe et al., 1996). Obtaining entire stomachs for dietary analysis is still a valid option for species targeted by fisheries because large numbers of samples can be collected over prolonged time frames (Gonzalez‐Pestana et al., 2021; Huveneers et al., 2007; Simpfendorfer et al., 2001a). However, for the vast majority of species not targeted for human consumption, it is imperative to consider ways of replacing lethal sampling altogether (Hammerschlag & Sulikowski, 2011).

Stable isotope analysis (SIA) has been used to study the trophic ecology of marine vertebrates since the late 1980s (Estep & Vigg, 1985; Harrigan et al., 1989). Since then, non‐lethal biochemical approaches, including SIA, have increased in elasmobranch trophic studies (Bornatowski et al., 2023; Petta et al., 2020). However, it is important to acknowledge that these methods answer different ecological questions and are not interchangeable (Hussey et al., 2012; Petta et al., 2020). With SCA, specific prey species or types can be identified either visually or with the aid of genetic verification (da Silveira et al., 2020). On the other hand, SIA generally provides dietary information at coarser taxonomic resolution and can lead to erroneous conclusions on relative prey importance if stable isotope compositions of prey overlap (Abrantes & Sheaves, 2024). Therefore, pairing SIA with SCA can overcome the limitations associated with each method individually, especially where little or no prior knowledge exists on the diet (Abrantes & Sheaves, 2024; Baker et al., 2024).

Given the feeding ecology of many shark and ray species remains poorly studied, incomplete or unknown, there is a continual need for SCA in ecological research. Gastric lavage presents a non‐lethal alternative to stomach dissection, which involves flushing the digestive tracts of live animals to assess prey consumption without killing them. It is generally performed using some type of pulsed water flow device to induce regurgitation or stomach inversion. The first records of this technique being applied to elasmobranchs were by Medved (1985) and Nelson and Ross (1992). Despite being successful in early studies, gastric lavage has not been widely used in subsequent decades. This is somewhat surprising since the procedure is relatively simple to perform, does not require specialist equipment and animals can be released afterward. Gastric lavage also remains more cost‐effective than other emerging techniques, such as identifying prey items from cloacal swabs with DNA metabarcoding (Clark et al., 2023; Olin et al., 2023).

Currently, information pertaining to the effectiveness of gastric lavage and survival of individuals is touched on across studies but has not been consolidated. In this study, we summarised gastric lavage studies on sharks and rays to identify which species/groups it has been effective for, the difficulties encountered and if post‐release survival has been assessed. Secondly, a field study is presented to demonstrate (1) how gastric lavage was performed on young juvenile rays, (2) its effectiveness at obtaining stomach contents and (3) post‐release survival over time using mark‐recapture methods.

## 2 MATERIALS AND METHODS

### 2.1 Literature summary

Published studies were searched in the Web of Science and Google Scholar databases from June to July 2024 using combinations of search terms including *gastric lavage*, *gastric evacuation*, *nonlethal sampling* and *stomach flush*, which were paired with *shark*, *ray* or *elasmobranch*. Studies were retained if the full text was available and if gastric lavage (or related term) was mentioned in the title, abstract or methods. The following details were then extracted: year published, field setting or captivity, purpose of study, species, shark or ray, life stages (neonate/young‐of‐year, juvenile/subadult or adult), sample size, gastric lavage efficiency and how post‐release survival was assessed. Note, to truly evaluate efficiency, animals would need to be dissected (unless stomach was inverted) to confirm without doubt that all contents were removed or if empty stomachs were actually empty. Thus, for the purpose of this study, efficiency represents the number/percentage of shark or rays sampled where stomach contents were collected.

### 2.2 Field study

Data collection took place at two intertidal sand flats in North Queensland, Australia. At both sites there are considerable changes in water depth across the tidal cycle, in which rays regularly migrate onto the upper intertidal zones during rising and high tides. Lucinda Beach (−18.5327°S, 146.3347°E) is an extensive intertidal flat on the border of the wet and dry tropics (full description of site provided by Crook et al. (2022)). Aside from the mangroves at the northern end, the study area is primarily unvegetated, with a mixture of coarse and fine sediments. The most commonly encountered ray species are the Australian whipray, *Himantura australis*, broad cowtail stingray, *Pastinachus ater*, and giant shovelnose ray, *Glaucostegus typus*, while brown whipray, *Maculabatis toshi*, and mangrove whipray, *Urogymnus granulatus*, are also present in smaller numbers (Crook, 2020; Myers, unpublished data). Rays were caught over 48 days from November 2022 to April 2024, and the number of days passed between consecutive site visits ranged from 1 to 107.

Blacksoil Creek (−19.299407°S, 147.042662°E) is an estuary inlet located at Cape Cleveland, approximately 112 km southeast of Lucinda Beach. The inlet is surrounded by saltpans, with little urban development or farmland in its immediate catchment (Mattone & Sheaves, 2017). The study area encompasses 0.5 km^2^ near the estuary mouth. During low tides, exposed sand flats cover most of the creek area, which are surrounded by narrow subtidal channels along the deeper mangrove edges. The most common species are *H. australis* and *M. toshi*. Although *G. typus* and *P. ater* are occasionally present, these were not targeted due to low occurrence. Rays were caught on 16 dates from February 2022 to June 2024, and the number of days passed between visits ranged from 1 to 293.

**FIGURE 1:**
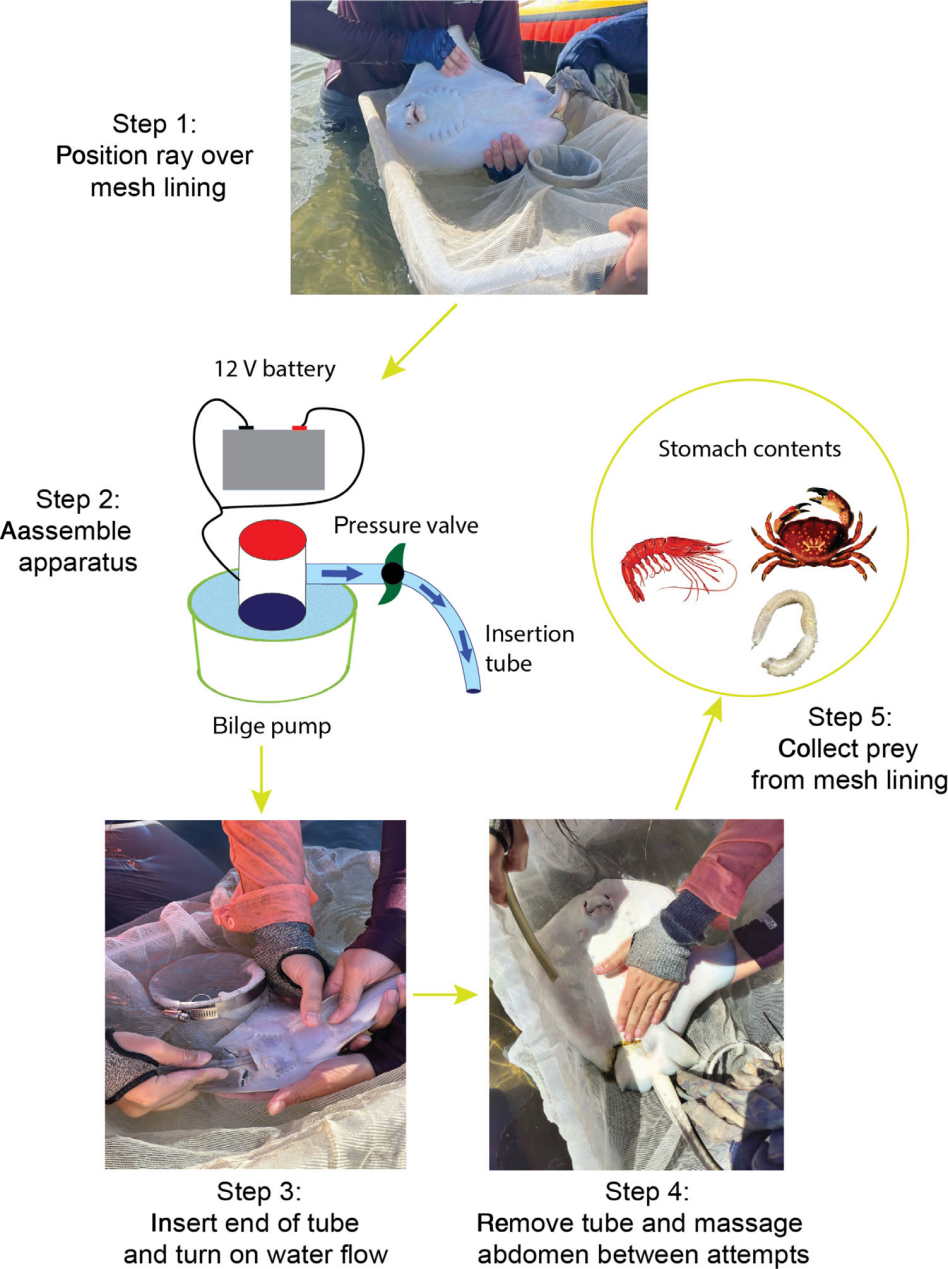
Diagram illustrating how to perform gastric lavage on small rays. The photographs in steps 3 and 4 demonstrate the procedure on *Glaucostegus typus* (step 3) and *Pastinachus ater* (step 4).

All rays were caught under general fisheries permit 259,152, with ethical approvals from James Cook University (Animal ethics approval 2838). Juvenile rays were captured in shallow water (<1 m) by encircling them in a beach seine net. Individual rays were then transferred to handheld dip nets and placed in a holding tray lined with 1 mm mesh netting. Gastric lavage procedures were adapted from Elston et al. (2020) and are illustrated in Figure 1. A 500 GPH capacity bilge pump was connected to a 12 V marine battery. The apparatus was fitted with an 8 mm diameter flexible plastic tube with a bevelled end, which was inserted into the mouth. A valve was fitted to the tube to adjust water pressure to the minimum amount needed to generate a firm, steady flow. The size of tube and flow rates were selected based on ray size, given that an oversized tube blocked materials from exiting the stomach and a tube that was too narrow did not generate sufficient water pressure. For all stingray species, the 8 mm tube was used on all individuals >50 cm disc width (DW), with an approximate flow rate of ~6.4 L/min. A smaller 6 mm tube was used on rays ranging from 30 to 50 cm DW (flow rate = ~4.4 L/min) and a 4 mm tube for rays <30 cm DW (flow rate = ~2.4 L/min). For *G. typus*, a 4 mm tube was used if the total length was <80 cm.

Gastric lavage was performed by two handlers. One person would grip the base of the stingray's tail with a gloved hand and invert the ray, supporting the dorsal side by placing a hand underneath the disc. The other handler would insert the bevelled end of the tube into the mouth by applying gentle pressure to bypass the oesophagus. The ray was then tilted with its head pointed downward to collect the regurgitated materials onto a mesh lining. Once water flow was initiated, flushing lasted between 10 and 15 s per attempt, with two or three attempts per individual. Once there was visual extension of the abdominal area, the tube was removed and the abdomen was lightly massaged forward towards the mouth. Prey items were expelled from either the mouth, gills or spiracles. Commonly during the procedure, faeces or digested materials from the lower gut were also pushed through the cloaca. Any expansion of the stomach and gut ceased once excess water was massaged out of the stomach. If no material was after the third attempt, the ray's stomach was assumed to be empty.

Total handling time ranged from ~5 to 15 min, which also included taking a muscle tissue sample from the posterior end of the disc and a clip from the pelvic fin for additional research objectives not in this study. A marker tag (numbered for identification) was inserted on the spiracles of *H. australis*, *M. toshi* and *P. ater* and on the dorsal fin for *G. typus*. Only the two largest *M. toshi* were given a marker tag, since all others were too small for attachment via the spiracle. Rays were released as close as possible to the capture location and were visually inspected for 2 min (unless they swam off) to ensure recovery. Generally, a ray either buried itself in the sediment or swam off immediately. No signs of injury or predation attempts were witnessed during the immediate recovery period. During repeated site visits, recaptured rays were identified by their marker tags. Gastric lavage was repeated if at least 3 days had passed from previous capture and the ray appeared in good condition (exhibiting normal behaviour, no remaining stress colouration, no deterioration in body condition).

Gastric lavage efficiency was represented by the frequency of occurrence (the percentage of stomachs that contained prey) and was calculated by dividing the total number of non‐empty stomachs by the total flushed stomachs × 100. This metric was calculated collectively for each species (pooling sites) and for species at each site. Here, we assumed that all stomachs were fully emptied with each flush and that an absence of stomach content was due to an empty stomach rather than poor technique or other external factors. The frequencies of successes (sample obtained) and failures (empty stomachs) were summarised by species (sites pooled) and by location for *H. australis* and *M. toshi* (Table S1), which were then compared using Fisher's exact tests. Where a significant result was obtained (*p* < 0.05), pairwise comparisons were run between specific pairs. For *G. typus*, frequencies and resulting gastric lavage efficiency were only calculated for rays caught in 2023, since *G. typus* caught in 2024 were used for multiple research objectives, where it was not consistently reported if an absence of stomach contents meant that gastric lavage was not performed or that an individual had an empty stomach. However, recaptured *G. typus* from 2024, for which contents were recorded (*n* = 11), were still included to assess post‐release survival. Moreover, frequency of occurrence and counts of specific prey types were recorded for future trophic analyses.

To assess post‐release recovery and survival of recaptured rays after gastric lavage, the total number of days between the first capture (when gastric lavage was performed) and last known recapture was recorded. This metric was omitted for individuals that shed their marker tag between the first and second captures because this made the initial capture date uncertain. Recapture data were summarised for each species to obtain the maximum length of time over which survival could be verified, the mean number of days between captures and to calculate recapture rates (%). Given that only three rays were recaptured from Blacksoil Creek (of which only two *H. australis* retained their marker tags), these recaptures were pooled with *H. australis* from Lucinda Beach.

## 3 RESULTS

### 3.1 Literature summary

**FIGURE 2:**
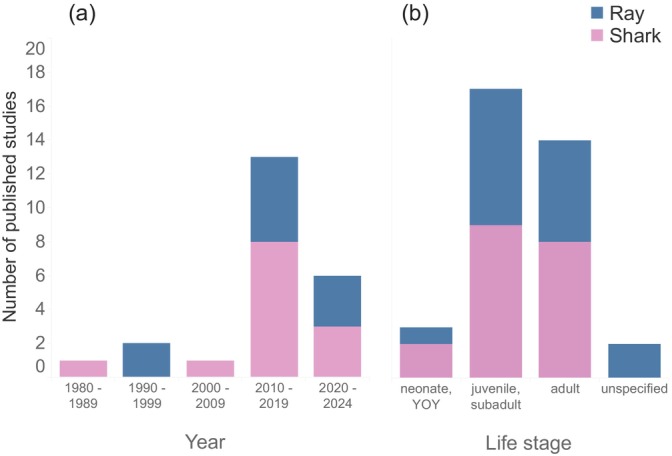
Histograms depicting number of published studies across decades (a) and the number of studies where each life stage was included (b). If multiple life stages were included, these studies were included for all applicable categories. Bar colour designates the number of studies for sharks and rays. YOY, young‐of‐year.

Excluding our field study, there were 23 published studies between 1985 and 2023 that reported using gastric lavage on either sharks or rays (Table 1). Only four studies were conducted before 2010, with the majority from 2010 to 2019 (Figure 2a). Studies mostly included later‐stage juveniles, subadults and adults, with only three studies featuring neonate or young‐of‐year age classes (Figure 2b). A total of 10 studies were conducted on rays and 13 on sharks, with 15 and 11 different species represented, respectively. The purposes of field studies (*n* = 15) were primarily to describe the diet of one or more species and to test gastric lavage efficiency, while captivity studies (*n* = 7) were designed to assess factors related to gastric evacuation times. Where sample sizes were reported, these ranged broadly from 15 to 336 individuals (Table 1). Size ranges indicated that most studies were done on small‐bodied shark species <200 cm total length, and the largest ray species included was the spotted eagle ray, *Aetobatus narinari* (reaching 187 cm DW). Gastric lavage efficiency ranged broadly from 29% to 100% for sharks and from 60% to 95% for rays.

**TABLE 1:** Summary of all studies that have reported using gastric lavage on sharks or rays.

Shark or ray	Study type	Study purpose	Species included	Life stage	Sample size by species	Size range or mean size (cm)	Gastric lavage efficiency (%)	Tube size (mm)	Main prey types recovered from field study or prey fed in captivity	How was recovery or survival assessed?	Reference
Shark	Captive	Gastric evacuation	*Carcharhinus plumbeus*	Juvenile	18	43–71	UNSP	UNSP	Crab, menhaden	Killed afterward	Medved (1985)
Ray	Captive	Gastric evacuation	*Raja erinacea*	UNSP	UNSP	33–51	UNSP	3	Polychaetes, crustaceans, sand lance, bivalves	Not assessed	Nelson & Ross (1992)
Ray	Captive	Gastric evacuation	*Raja erinacea*	UNSP	UNSP	33–51	UNSP	3	Polychaetes, krill, sand lance, bivalves	Monitored in captivity	Nelson & Ross (1995)
Shark	Captive	Gastric evacuation	*Sphyrna lewini*	Juvenile	64	50–60	UNSP	UNSP	Herring	Monitored in captivity	Bush & Holland (2002)
Shark	Field	Diet	*Notorynchus cepedianus*	Subadult, adult	336	150–290	54	30	Sharks, teleosts, rays and mammals	Not assessed	Barnett, Abrantes, et al. (2010)
Shark	Field	Diet with DNA verification, efficiency	*Notorynchus cepedianus*	Subadult, adult	100	150–290	50	30	Sharks, teleosts, rays and mammals	Tag‐recapture, acoustic tracking	Barnett, Redd, et al. (2010)
Ray	Field	Diet	*Glaucostegus typus*, *Himantura fai*, *Himantura uarnak*, *Pastinachus atrus*, *Himantura astra/toshi*	Juvenile, adult	74, 46, 20, 10, 8	UNSP	69, 80, 69, 60, 75	20	Crustaceans, polychaetes for *P. atrus* only	Not assessed	Vaudo & Heithaus (2011)
Ray	Field	Diet	*Aetobatus narinari*	Juvenile, adult	18	99–170	78	UNSP	Bivalves	Not assessed	Ajemian et al. (2012)
Shark	Field	Diet	*Squalus acanthias, Mustelus antarcticus*	Juvenile, subadult, adult	139, 136	25–73, 60–140	55, 92	UNSP	Teleosts, cephalopods, crustaceans	Not assessed	Yick et al. (2012)
Shark	Field	Efficiency	*Squalus acanthias*	Juvenile, adult	45	84	89	20–37	Mixed invertebrates, teleosts	Killed afterwards	Bangley et al. (2013)
Shark	Captive	Gastric evacuation	*Squalus acanthias*	Adult	15	98	UNSP	37	Menhaden	Monitored in captivity	Bangley & Rulifson, 2014
Ray	Field	Efficiency	*Urogymnus asperrimus*	Juvenile	55	43–81	95	14.4	Annelids, crustaceans	Visual inspection after release	Elston et al. (2015)
Ray	Captive	Gastric evacuation	*Leucoraja eglanteria*	Subadult, adult	77	57–73	UNSP	No tube used	Sand lance	Not assessed	Stehlik et al. (2015)
Shark	Field	Diet	*Carcharhinus amblyrhynchos, Carcharhinus melanopterus, Triaenodon obesus*	Adult	31, 45, 31	68–158	32, 58, 29	20	Teleosts	Not assessed	Frisch et al. (2016)
Ray	Field	Diet	*Urobatis jamaicensis*	Juvenile, adult	117	18	77	10	Polychaetes, prawns	Visual inspection after release	O'Shea et al. (2018)
Shark	Field	Diet	*Galeocerdo cuvier*	YOY	UNSP	UNSP	UNSP	UNSP	Birds, other contents not reported	Not assessed	Drymon et al. (2019)
Shark	Field	Diet	*Carcharhinus melanopterus*	Neonate, juvenile	274	29–47 LPC	62	25–38	UNSP	Not assessed	Weideli et al. (2019)
Ray	Field	Diet	*Pastinachus ater*, *Urogymnus granulatus*	Juvenile	50, 39	28–140	71, 78	14.4	Bivalves, crustaceans	Visual inspection after release	Elston et al. (2020)
Ray	Field	Diet	*Styracura schmardae*, *Hypanus americanus*	Juvenile, adult	74	68, 67	64	10	Crustaceans, annelids	Not assessed	O'Shea et al. (2020)
Shark	Captive	Gastric evacuation	*Squalus acanthias*	Adult	15	81–93	UNSP	50	Sand lance	Monitored in captivity	Stehlik et al. (2021)
Shark	Field	Diet	*Triakis semifasciata*	Juvenile, adult	30	40–139	100	No tube used	Annelids, mixed invertebrates, plant matter	Not assessed	Cooper (2022)
Ray	Field	Diet with DNA verification	*Aetobatus narinari*	YOY, juvenile, adult	61	59–187	82	9.5–15.8	Bivalves	Not assessed	Cahill et al. (2023)
Shark	Field	Diet	*Negaprion acutiden*, *Carcharhinus melanopterus*	Juvenile	115, 188	55, 48 PCL	46, 79	25–38	Teleosts	Not assessed	Weideli et al. (2023)
Ray	Field	Diet, efficiency	*Glaucostegus typus*, *Himantura australis*, *Maculabatis toshi*, *Pastinachus ater*	Neonate, YOY, juvenile	83, 47, 34, 63	30–115, 26–76, 18–35, 30–68	81, 80, 94, 71	4–8	Crustaceans, polychaetes and molluscs for *P. ater only*	Tag‐recapture	Current study

*Note*: Details were pooled for all studies that included data from multiple sites. For sharks, sizes are given in centimetres as total length unless otherwise specified. For rays, size is reported as the disc width, except for *Glaucostegus typus*, which used total length. For studies with multiple species, the sample sizes, size ranges and percentage of non‐empty stomachs are reported for each species in the order listed.

Abbreviations: LPC, length to caudal peduncle; PCL, precaudal length; UNSP, any characteristic that was unspecified within a study; YOY, young‐of‐year.

Although multiple studies confirmed short‐term recovery by visual inspection after release, only one study assessed longer‐term survival, therefore evidence of survival was mostly derived from captivity settings. Survival was verified for days or weeks after gastric lavage for shark species including spiny dogfish, *Squalus acanthias*, and scalloped hammerhead, *Sphyrna lewini*, as well as two species of skates, the clearnose skate, *Leucoraja eglanteria*, and the little skate, *Raja erinacea*. Where individuals were not killed immediately afterward to verify gastric evacuation rates, authors reported using the same individuals for other experiments or releasing them. No studies reported mortality or ill‐effects during or directly after gastric lavage.

### 3.2 Field study

A total of 209 rays were sampled using gastric lavage, which included 43 from Blacksoil Creek and 166 from Lucinda Beach (Table 2). Including recaptured rays, gastric lavage was used 243 times. Observed size ranges confirmed all rays to be juveniles across multiple age classes. A broader size range of *H. australis* was caught at Blacksoil Creek (30–76 cm disc DW) than Lucinda Beach (26–51 cm DW), while sizes of *M. toshi* were similar across sites, ranging from 18 to 35 cm DW overall. Gastric lavage efficiency was 83.3% for *H. australis* and 100% for *M. toshi* at Blacksoil Creek. For Lucinda Beach, percentages were 71.4% for *P. ater*, 78.1% for *H. australis*, 81.8% for *M. toshi* and 80.7% for *G. typus* (Table 2). Gastric lavage efficiency showed marginal variation due to species (Fisher's exact test, *p* = 0.051), while no differences were observed between sites (*p* = 0.1343). Across both sites, 31 samples were obtained from recaptured rays, including nine *H. australis*, one *M. toshi*, five *P. ater* and 16 *G. typus*. Notably, there were two *H. australis* individuals for which three samples were obtained over time. The first, which was captured at Blacksoil Creek, was recaptured twice within 12 weeks (~4 weeks between attempts). The other was caught three times at Lucinda Beach, where ~30 days passed between attempts.

**TABLE 2:** Gastric lavage summary for *Himantura australis*, *Maculabatis toshi*, *Pastinachus ater* and *Glaucostegus typus* caught at Blacksoil Creek and Lucinda Beach.

Site	Species	Number of rays sampled by gastric lavage (excluding recaptures)	Number of rays sampled by gastric lavage (including recaptures)	Number of non‐empty stomachs (including recaptures)	Gastric lavage efficiency (%)	Number of recaptured rays	Number of samples obtained from recaptured rays	Recapture rate (%)	Mean size ± SE (cm)	Size range (cm)
Blacksoil Creek	*H. australis*	21	24	20	83.3	2	3	9.5	49.8 ± 3.6	30–76
	*M. toshi*	22	23	23	100.0	1	1	–	24.8 ± 0.7	20–35
Lucinda Beach	*H. australis*	26	32	25	78.1	5	6	19.2	37.9 ± 1.3	26–51
	*M. toshi*	11	11	9	81.8	0	0	–	25.7 ± 1.3	18–34
	*P. ater*	64	70	50	71.4	15	5	23.4	39.5 ± 0.6	30–68
	*G. typus* (2022–2023)	48	57	46	80.7	23	16	33.8	45.9 ± 1.0	30–115
	*G. typus* (2024)	17	26	–	–					

*Note*: From the 2024 dataset, it was known that at least 26 *G. typus* were sampled since stomach content samples were obtained. –, denotes that this the number of flushes resulting in empty stomachs was unknown and gastric lavage efficiency was not calculated. Recapture rate refers to the percentage of the total that were recaptured over the study. This measure was omitted from *M. toshi* because most individuals were too small to be given a spiracle marker tag.

Seven recaptured rays (six *G. typus*, one *M. toshi*) shed their marker tags over the study period, which was evident by scar tissues on the spiracle or dorsal fin where the tag was fitted. Days between recaptures were not calculated for these individuals. Recapture rates were unknown for *M. toshi* because most individuals were too small to be tagged. Only one recapture at Blacksoil Creek was recorded with uncertainty, based on scar tissue on its disc from tissue sampling. Excluding these individuals, recaptures of *G. typus*, *H. australis* and *P. ater* provided evidence of short‐ and longer‐term survival after handling. Recapture rates for each species at Lucinda Beach were 33.8%, 19.2% and 23.4%, respectively, although only 9.5% of *H. australis* were recaptured at Blacksoil Creek (Table 2). It was also common for individuals to be recaptured more than once (seven *G. typus* and one *H. australis* at Lucinda Beach, one *H. australis* at Blacksoil Creek).

**FIGURE 3:**
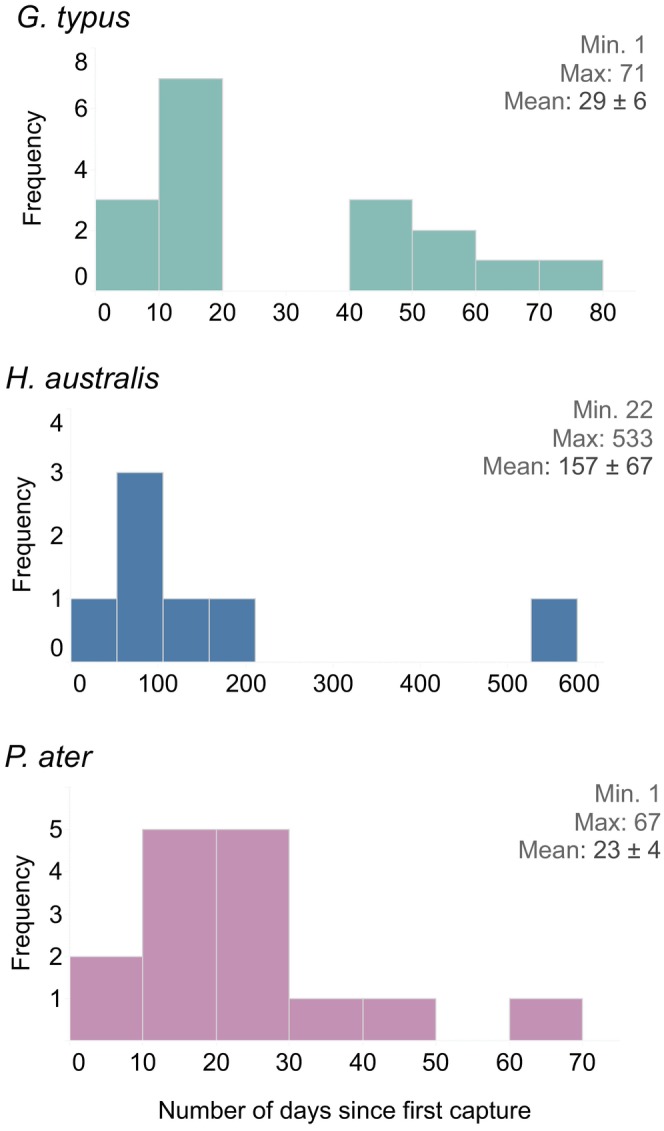
Histogram illustrating the number of days that passed between first and last recaptures of *Glaucostegus typus*, *Himantura australis* and *Pastinachus ater*.

Although 23 *G. typus* were recaptured at Lucinda Beach, the number of days between captures was only calculated for 17 rays due to tag loss (Figure 3). Recaptures ranged from 1 to 67 days following gastric lavage, with a mean of 29.1 ± 23.3 standard deviation (SD). For *P. ater* (*n* = 15), recaptures occurred from 1 to 71 days (mean = 23.1 ± 16.2 SD). Across both sites, recaptures were less frequent for *H. australis* (*n* = 7) and spanned a highly variable range of 22–533 days (mean = 157 ± 176.5 SD; Figure 3). Visual assessments did not reveal any rays with notable decreases in body condition, extended abdominal cavity, bruising or abnormal colourations or markings. The exception to this was that some rays recaptured within 1–2 days had lingering pink colouration on the underside of the disc, which could be an indicator of stress.

## 4 DISCUSSION

### 4.1 Trends in gastric lavage use

Gastric lavage (stomach/gut flushing) has long been established as an alternative to lethal stomach dissection for collecting stomach contents of marine vertebrates including fish (Kamler & Pope, 2001), sea turtles (Forbes & Limpus, 1993) and marine mammals (Antonelis Jr et al., 1987). Notably, only 23 published studies have used this technique on elasmobranchs, and its use has only modestly increased since 1985, despite growing demands for non‐lethal SCA. However, some trends emerge when summarising its use across studies. Gastric lavage has been predominantly used on smaller‐bodied sharks or rays, which may be because it is challenging or impossible to perform on animals that cannot be boarded onto a vessel or manually lifted and positioned over a collection tray. Notably, *N. cepedianus* is the largest species for which gastric lavage has been used (maximum 296 cm total length [TL]), which were brought onto a vessel for sampling (Barnett, Abrantes, et al., 2010; Barnett, Redd, et al., 2010). No studies have reported flushing larger animals while in the water and restrained to the side of a vessel.

Although not explicitly mentioned, there may also be concerns about gut flushing causing internal damage (particularly for developing individuals), which may result in low numbers of studies on neonates and young‐of‐year life stages. However, elasmobranchs have robust stomachs that can even be inverted and pushed through their mouths (Brunnschweiler et al., 2005; Cortés & Gruber, 1990). Anecdotal reports have also revealed dissected sharks with several stingray barbs puncturing their stomachs, which further suggests their resilience to internal damage (M. Braccini, personal communication, A. Barnett, communication with several fisherman). Although shark and ray morphology appears well suited for gastric lavage, species physiology and behaviour must also be considered when assessing its use. For example, it may not be suitable for species that are highly vulnerable to capture and handling stress, such as larger hammerhead sharks (Sphyrnidae) (Gallagher et al., 2014; Jerome et al., 2018).

Gastric lavage seems to be an effective method of extracting the stomach contents of sharks and rays, with success rates often exceeding 50% (Table 1). However, sample sizes varied considerably, which may affect our ability to generalise findings across studies. One notable pattern was that gastric lavage was more successful at extracting stomach contents from rays than sharks. The results of our field study coincided with this result, in which efficiency was high for all four ray species. The small size ranges for each species indicated that most individuals were either neonate or young‐of‐year size classes, based on known age‐growth relationships (Gaskins et al., 2020; Last et al., 2016; O'Shea, Braccini, et al., 2013). Rates were comparable to studies that used similar methodologies on juvenile ray species, including *U. asperrimus* (95%), *P. ater* (71%) and *U. granulatus* (78%) (Elston et al., 2015, 2020). One reason for higher success in benthic rays may be related to feeding frequency. Stingrays are known to be continuous feeders (Gilliam & Sullivan, 1993; Jacobsen & Bennett, 2013), which may be because their prey are easy to acquire, being predominantly benthic and slow‐moving. In contrast, larger sharks that target faster‐moving pelagic prey may feed less frequently due to lower encounter and catchability (Wetherbee et al., 2004). Another factor is that sharks, particularly species like sevengill sharks and tiger sharks, have been observed to regurgitate or “evert” their stomachs when hooked, which could result in empty stomachs (Barnett, Redd, et al., 2010; Simpfendorfer et al., 2001b). However, this limitation is not exclusive to non‐lethal methods because high rates of empty stomachs are also common in stomach dissections (Bethea et al., 2004, 2006).

Variable success was likely driven by several other factors that are unique to the context of each study. For instance, the likelihood of obtaining stomach contents may depend on differences in stomach morphologies (Waters et al., 2004) or animal size (Bangley et al., 2013; Cailteux et al., 1990; Weideli et al., 2019). Additionally, for animals with broad diets, prey types with specific morphologies may be more easily dislodged from the stomach than others. For example, Cahill et al. (2023) admitted there was a higher dominance of gastropods in the stomachs of *A. narinari* caught by commercial fisheries (Serrano‐Flores et al., 2019) than those sampled by gastric lavage, likely because these items were not as easily extracted during flushing. Success may also depend on whether sampling aligns with peak feeding times. For example, many reef sharks exhibit crepuscular hunting behaviours, so sampling during the day could result in over‐digested prey or empty stomachs (Hammerschlag et al., 2017). In our study, sampling during times when rays migrate into intertidal zones to feed may have increased the likelihood of encountering freshly consumed prey within the stomachs.

Studies also varied by equipment (e.g. flexible tubing vs. PVC pipe) and flushing techniques (pulsed vs. continuous). When optimising protocols for our field study, a major consideration was selecting appropriately sized tubes relative to the size of the animal. Bangley et al. (2013) found that differences between mouth width and tube diameter had the greatest impact on gastric lavage efficiency and recommended that the tube should be no more than 10–20 mm smaller than the mouth diameter. Methods by Elston et al. (2015) described extracting stomach contents from *U. asperrimus* (43–81 cm DW) with a 14.4 mm tube. A study by Vaudo and Heithaus (2011) also performed gastric lavage on *G. typus*, *H. uarnak*, *M. toshi* and *P. ater* with a 20 mm diameter tube. As most individuals included here were smaller than in previous studies, both tube sizes would have been unsuitably large, particularly for young‐of‐year *M. toshi* (generally <25 cm DW) and *G. typus* (<50 cm TL). We therefore trialled a range of tube diameters (4, 6 and 8 mm) and established guidelines for which sizes were most appropriate for different size classes. This optimisation procedure was also done by Cahill et al. (2023) on *A. narinari* and by Weideli et al. (2019) on early developmental stages of *C. melanopterus*.

Relationships between tube size and lavage efficiency were not tested experimentally in our field study, but early attempts indicated that higher success was achieved with narrower tubes, which may prevent the oesophagus from being completely obstructed during flushing. Water pressure also had to be adjusted to avoid overexpanding the stomach. Flow rates varied based on tube size so these were manually adjusted before each gastric lavage attempt. Although it was normal for the abdomen to expand during flushing, expansion would subside once the tube was removed or after excess water and trapped air were massaged from the abdomen towards the cloaca. There were no visual indicators of internal damage, although this could have only been verified by dissecting individuals directly following lavage (Bangley et al., 2013).

Although gastric lavage can be effective for obtaining stomach contents, some additional limitations may inhibit its adoption by researchers. A well‐voiced concern is that not all items may be evacuated from the gut, leading to underestimations of the total prey consumed or erred estimates of nutritional contributions. Some studies addressed this concern by dissecting a subsample of individuals after flushing (Ajemian et al., 2012; Cooper, 2022; Frisch et al., 2016; Stehlik et al., 2015). As each of these confirmed that stomachs were effectively emptied, results would not be expected to vary between gastric lavage and stomach dissection. Although skipping this step means we cannot fully eliminate these assumptions, killing a subset of individuals in every study may not be desirable or practical. Although gastric lavage has been performed on relatively few species overall, these data can still be compared to stomach dissection studies to validate its efficiency. For instance, Barnett, Abrantes, et al. (2010) found that gastric lavage was similarly effective to stomach dissection for *N. cepedianus*. Furthermore, the results of our field study were comparable to the successes obtained using stomach dissections on similar species (O'Shea, Thums, et al., 2013). Another limitation for any method of SCA is that overly digested stomach contents can influence accurate identification and counts of individual prey items, and potentially unemptied stomachs and unidentifiable materials create uncertainty for common dietary metrics based on prey abundance, volume or weight. However, since there are several issues when basing dietary habits on volume or weight (Amundsen & Sánchez‐Hernández, 2019; Baker et al., 2014), more robust metrics, such as frequency of occurrence, are now being advocated to generalise diets (Baker et al., 2024).

Verifying survival after invasive sampling procedures is important for developing best practice protocols and justifying methods are truly non‐lethal. Captivity studies provided the most information on animal wellbeing and survival after handling, since the behaviours and body condition of animals can be visually monitored for prolonged periods. No ill‐effects from gastric lavage were reported on captive animals, and in some cases animals were re‐used or released following a recovery period of days or weeks (Bangley & Rulifson, 2014; Nelson & Ross, 1995). Verifying survival in the field is challenging because most animals can only be visually observed for a short window of time. Subsequently, field studies have either not reported any information on recovery or survival or have only done so immediately after release (Ajemian et al., 2012; Elston et al., 2015, 2020; O'Shea et al., 2020). To our knowledge, only one field study has assessed longer‐term survival following gastric lavage by implementing a combination of tag‐recapture and acoustic tracking methods (Barnett, Redd, et al., 2010). In this study, *N. cepedianus* were recaptured over a span of 11–715 days, and all 20 sharks fitted with acoustic tags were detected within the array up to 18 months later, which were both strong indicators of high survival.

Tag‐recaptures from our field study also provided evidence of survival for juvenile rays across various time frames post‐release. Most recaptures occurred within days or weeks of the first capture, except for one *H. australis* at Blacksoil Creek that was recaptured 533 days later. There was one case where a tagged juvenile *G. typus* at Lucinda Beach was reported dead 6 weeks after capture, but with the amount of time that passed, there was no conclusive evidence that this event was linked to handling. No other mortality events are known to have occurred. Shark recaptures are often <20% (Dudgeon et al., 2015) or even as low as 5% (Kohler & Turner, 2001). However, similar recapture rates (<20%) have been reported for ray species such as the blue‐spotted lagoon ray, *Taeniura lymma* (McIvor et al., 2024), which match our results for *H. australis*. Furthermore, other studies report similar results as our recapture rates for *G. typus* (33.8%) and *P. ater* (23.4%). For example, Schwanck et al. (2020) reported 31% of southern stingrays, *Hypanus americanus*, were recaptured in sand flats, cays and creeks, whereas O'Shea et al. (2021) also reported 51% for juvenile Caribbean whiptail stingrays, *Styracura schmardae*, within tidal creek nurseries.

The relatively high recapture success in this field study may be attributed to the predictable movements of juvenile rays across intertidal flats over repeated tidal cycles (Crook, 2020; Martins et al., 2020), which increased the probability of encountering tagged individuals within the study area. The main limitation of tag‐recapture data is assuming that the survival of recaptured animals is representative of animals that are never recaptured throughout a study. Additionally, there may be issues with tag retention (Pine et al., 2012). Tag loss was more common for *G. typus* than other species because external tags were more easily shed from the dorsal fin than from the spiracles. Although recaptured individuals could still be identified by scar tissue on the dorsal fin, it was impossible to estimate how many days had passed since initial capture, which reduced our ability to quantify survival over longer time frames.

### 4.2 Conclusions

Understanding a predator's dietary composition is fundamental for evaluating its influence on food web dynamics and ecosystem function, which strengthens our ability to identify and protect essential habitats based on resource availability (Barnett et al., 2013, 2017; Heupel et al., 2014). Gastric lavage is an effective approach for extracting stomach contents from a variety of shark and ray species but remains underutilised in field research, and several methodological refinements warrant further investigation. The current study demonstrates several adaptable features that make this technique particularly effective for juvenile rays, including the use of smaller diameter tubes (4–8 mm) and a valve to control water pressure based on animal size, which was critical for successfully sampling neonate and young‐of‐year rays while minimising stress and potential injury. These methodological refinements provide a framework for expanding gastric lavage across different size classes and species, although additional research is still needed to adapt the necessary protocols (e.g. tube size, water pressure, handling techniques) for larger sharks, where specimen handling presents unique challenges.

Despite gastric lavage being acclaimed as non‐lethal, there is little evidence of recovery and survival for wild‐caught animals, aside from verifying the condition of individuals at the time of release. A paucity of data from field‐based studies results in survival being almost exclusively based on captive studies. Where possible, future studies should consider methods that integrate survival data into their research objectives. Monitoring animals over time using active tracking, acoustic arrays or satellite positioning tags are all appropriate methods for verifying post‐release survival but may not be practical due to cost, time constraints or the size of the target species. Although recapture rates of elasmobranchs are generally low, tag‐recapture represents a practical method and may be particularly suitable for specific contexts, such as juvenile animals within defined nursery grounds (as in our field study) or species with predictable movement or aggregation patterns. Future studies could also reduce tag loss by using internal tags (e.g. Passive Integrated Transponder [PIT] tags), which would allow for more precise tracking of individuals over extended periods. Furthermore, tag‐recapture can be supplemented by other methods such as electronic tracking, photo ID or genetics (Dudgeon et al., 2012; McIvor et al., 2024). For smaller species, studies also consider including a survival component in captivity. Validating survival following gastric lavage (or any non‐lethal handling procedure) will ensure that protocols comply with ethical standards and ensure the best outcomes for research and conservation.

## AUTHOR CONTRIBUTIONS

Jaelen Myers: Conceptualisation, investigation, writing – original draft, formal analysis. Marcus Sheaves: Supervision, writing – review and editing. Adam Barnett: Conceptualisation, supervision, writing – review and editing.

## Supporting information


**TABLE S1.** Contingency table showing the frequencies of successful (non‐empty stomachs) versus unsuccessful (empty stomachs) gastric lavage outcomes by species (sites pooled for *Himantura australis* and *Maculabatis toshi*) and by location.
