# Supplementary material for: Summarising 40 years of gastric lavage studies to evaluate efficiency and survival in sharks and rays
Source: J Fish Biol. 2025 Mar 4;107(1):130–42. doi: 10.1111/jfb.70006 (PMC12327170; doi:10.1111/jfb.70006)
Supplement: Supplementary file 1 — TABLE S1. Contingency table showing the frequencies of successful (non‐empty stomachs) versus unsuccessful (empty stomachs) gastric lavage outcomes by species (sites pooled for Himantura australis and Maculabatis toshi) and by location. [file JFB-107-130-s001.docx]

Supporting information

Table S1. Contingency table showing the frequencies of successful (non-empty stomachs) versus unsuccessful (empty stomachs) gastric lavage outcomes by species (sites pooled for *Himantura australis* and *Maculabatis toshi*) and by location.

| Site and species | No. containing stomach contents | No. empty stomachs |  |
| --- | --- | --- | --- |
| Both sites |  |  |  |
| *G. typus* | 46 | 11 |  |
| *H. australis* | 45 | 11 |  |
| *M. toshi* | 32 | 2 |  |
| *P. ater* | 50 | 20 |  |
|  |  |  |  |
| Blacksoil Creek |  |  |  |
| *H. australis and M. toshi* | 43 | 4 |  |
|  |  |  |  |
| Lucinda Beach |  |  |  |
| *H. australis and M. toshi* | 34 | 9 |  |
|  |  |  |  |
|  |  |  |  |
